# Effectiveness and safety of tenofovir alafenamide/emtricitabine/bictegravir as a first-line regimen in people with HIV: A retrospective observational study

**DOI:** 10.1016/j.ijregi.2025.100622

**Published:** 2025-03-06

**Authors:** Andrea Giacomelli, Maria Vittoria Cossu, Davide Moschese, Giorgia Carrozzo, Serena Reato, Federico Sabaini, Giacomo Pozza, Martina Laura Colombo, Chiara Fusetti, Anna Lisa Ridolfo, Cristina Gervasoni, Spinello Antinori, Andrea Gori

**Affiliations:** 1 Department of Biomedical and Clinical Sciences, Università degli Studi di Milano, Milan, Italy; 2 III Division of Infectious Diseases, ASST Fatebenefratelli Sacco, Luigi Sacco Hospital, Milan, Italy; 3 Infectious Diseases Unit, ASST Fatebenefratelli Sacco, Luigi Sacco Hospital, Milan, Italy; 4 Centre for Multidisciplinary Research in Health Science (MACH), University of Milano, Milano, Italy 5II Infectious Diseases Unit, ASST

**Keywords:** HIV, Bictegravir, Single-tablet regimens, Advance naïve, Durability

## Abstract

**Objectives:**

To assess the effectiveness and safety of tenofovir alafenamide/emtricitabine/bictegravir (TAF/FTC/BIC) in patients newly diagnosed with HIV (PWH) in a non-experimental setting.

**Methods:**

We conducted a single-center, retrospective observational study that included all newly diagnosed PWH treated with TAF/FTC/BIC at our institution. Virological failure was defined as two consecutive HIV-RNA values of >50 cp/ml after 48 weeks of treatment. Reasons for TAF/FTC/BIC interruption were also collected. The durability of TAF/FTC/BIC was estimated using Kaplan-Meier curves.

**Results:**

A total of 236 PWH started TAF/FTC/BIC, with a median follow-up time of 13 months (interquartile range [IQR] 4-27 months). Most PWH were cisgender men (178/236, 75.4%) with a median age at diagnosis of 37 years (IQR 29-48) and a median cluster of differentiation 4 cell counts of 302 cells/mm³ (IQR 117-467). One protocol-defined virological failure was observed, without the development of drug resistance, resulting in an incidence of 3.1 per 1000 person-years of follow-up (95% confidence interval [CI] 0.8-17.3). Six (2.5%) PWH discontinued TAF/FTC/BIC because of toxicity. The estimated durabilities of TAF/FTC/BIC at 12 and 24 months were 84.8% (95% CI 78.6-89.3%) and 75.5% (95% CI 67.6-82.6%), respectively.

**Conclusions:**

In our cohort of newly diagnosed PWH treated with TAF/FTC/BIC, the low occurrence of virological failure and discontinuation related to drug toxicities underscores the effectiveness and tolerability of the regimen.

## Highlights

• Tenofovir alafenamide/emtricitabine/bictegravir (TAF/FTC/BIC) is recommended by international guidelines as a first-line regimen.
• TAF/FTC/BIC showed low virological failure in newly diagnosed people with HIV.
• Estimated TAF/FTC/BIC durability was 84.8% at 12 months, 75.5% at 24 months.
• Few toxicities led to TAF/FTC/BIC interruptions among the study cohort.

## Introduction

Integrase strand transfer inhibitor (INSTI)-based regimens are recommended by international guidelines as first-line options for people with HIV (PWH) [1,2]. This recommendation is based on the efficacy and safety profile of INSTI-based regimens demonstrated in randomized clinical trials (RCTs) [[3], [4], [5], [6]]. Additionally, second-generation INSTIs [i.e., dolutegravir and bictegravir (BIC)] have shown a high barrier to the development of drug resistance [[3], [4], [5], [6], [7]].

Tenofovir alafenamide/emtricitabine/BIC (TAF/FTC/BIC) is a single-tablet regimen that has demonstrated high efficacy, a high genetic barrier to resistance, low potential for drug-to-drug interaction, and a good safety profile in RCTs [[3], [4], [5], [6]]. Furthermore, TAF/FTC/BIC has been studied in various clinical scenarios, both in newly diagnosed PWH as a first-line regimen and in the context of antiretroviral treatment optimization as a switch strategy [[8], [9], [10], [11], [12]].

The initiation of antiretroviral treatment in a newly diagnosed PWH is a crucial step, with the priority being to not jeopardize future therapeutic options [1,2]. This necessity must be combined with the requirement for a rapid start of antiretroviral treatment, usually pending the results of baseline resistance tests [13]. Italy is among the countries in Western Europe with the highest proportion of PWH diagnosed late and treatment outcome real-world data on this special population are crucial to assess the effectiveness in a non-experimental context [14,15]. In this setting an antiretroviral treatment that is potent and has a low potential for drug-to-drug interaction, often necessitated by the need for co-medications in this setting, is preferred [16]. Nevertheless, late presenters (<350 cells/mm^3^) are underrepresented in regulatory randomized clinical trials (RCTs) which are not usually designed to assess this population. To the best of our knowledge, one RTC is currently underway in this context comparing TAF/FTC/BIC vs darunavir-based three-drug regimens [17]. Thus, data from clinical practice providing information in a real-world context characterized by a high proportion of PWH presenting late at the time of the first antiretroviral start could be useful for understanding the performance of TAF/FTC/BIC. We aimed to assess the effectiveness and safety of TAF/FTC/BIC in newly diagnosed PWH and identify reasons for discontinuation in a non-experimental context.

## Materials and methods

### Study design

This was a single-center retrospective observational study.

### Setting

The study was conducted at the Infectious Disease Department (Luigi Sacco Hospital, Milan, Italy).

### Study population

All PWH with a new HIV diagnosis between August 1, 2019, and February 7, 2024. PWH who received a prior antiretroviral regimen other than TAF/FTC/BIC were excluded. Subjects were followed until TAF/FTC/BIC interruption, death, administrative censoring, or March 25, 2024, whichever occurred first.

### Data collection

Medical records were reviewed to collect data on demographics, HIV-related parameters, treatment duration, adverse events, and reasons for discontinuation. PWH was categorized in accordance with cluster of differentiation (CD4) cell nadir strata (< *vs* ≥350 cell/mm^3^ and < *vs* ≥200 cell/mm^3^) and according to HIV-RNA zenith (< *vs* ≥ 500,000 copies/ml). Reasons for TAF/FTC/BIC interruption were collected and categorized as simplification, death, drug-to-drug interaction, toxicity, pregnancy, virological failure, and enrollment in a randomized clinical trial. Virological failure was defined as two consecutive HIV-RNA >50 cp/ml after 48 weeks from treatment start.

### Outcomes

The primary outcome was the occurrence of protocol-defined virological failure.

The secondary outcome was the assessment of reasons for treatment discontinuation and the durability of the regimen.

### Statistical analysis

The occurrence of virological failure was estimated by analyzing individuals with an adequate follow-up (treatment started before February 2023) and expressed by incidence rate normalized per 1000 person-years of follow-up with a 95% confidence interval (CI) calculated using Poisson distribution.

The durability of TAF/FTC/BIC was estimated using Kaplan-Meier curves and the durability according to biological sex (men vs women), age (< vs ≥60 years), and CD4 cell count (< *vs* ≥350 cell/mm^3^ and < *vs* ≥200 cell/mm^3^) and AIDS presentation were assessed using log-rank test.

Statistical analysis was performed using StataNnow/18.5 MP edition.

### Ethical statement

The study was approved by the institutional ethics committee comitato etico (CET) Lombardia 1 (CET 218-2024) and complied with the Declaration of Helsinki and good clinical practice guidelines.

## Results

### Study population

During the study, period 236 PWH started TAF/FTC/BIC as the first-line regimen with a median time of observation of 13 months (interquartile range [IQR] 4-27 months). Most PWH were cis men (178/236, 75.4%), 21 (8.9%) were transgender women and the remaining 37 (15.7%) were cis women with a median age at diagnosis of 37 years (IQR 29-48). The characteristics of the study population are reported in Table 1. The main mode of HIV acquisition was via homosexual intercourse (107/236, 45.3%) followed by heterosexual contact (71/236, 30.1%). The median CD4 cell count at diagnosis was 302 cells/mm^3^ (IQR 117-467). Ninety (38.1%) individuals presented with a CD4 cell count <200 cell/mm^3^ and 64 (27.1%) with an AIDS-defining condition and 30 (12.7%) with an HIV-RNA count >500,000 cp/ml.

**Table 1:** Characteristic of the study population.

Characteristics of the study population	Overall (n = 236)
Male sex at birth, n (%)	199 (84.3)
Age at diagnosis, median years (interquartile range)	37 (29-48)
Mode of HIV acquisition, n (%)	
Men who have sex with men	128 (45.3)
Heterosexual	71 (30.1)
People who inject drugs	7 (3.0)
Not reported	30 (12.7)
Ethnicity, n (%)	
Caucasian	151 (64.0)
African	15 (6.4)
Hispanic	65 (27.5)
Asian	5 (2.1)
AIDS-defining condition, n (%)	64 (27.1)
HIV-RNA > 500.000 cp/ml at ART start, n (%)	30 (12.7)
CD4 < 350/mmc at ART start, n (%)	126 (53.4)
CD4 <200/mmc at ART start, n (%)	64 (27.1)
Coinfections, n (%)	
Hepatitis C virus antibody-positive	14 (5.9)
Hepatitis B surface antigen-positive	6 (2.5)

ART, antiretroviral therapy; CD, clusters of differentiation; n, number.

### Virological failure, survival analysis, and reasons for discontinuation

One (0.4%) individual out of 221 PWH with an adequate follow-up contributing for 322 person-years experienced a protocol-defined virological failure accounting for an incidence rate of 3.1 per 1000 person-years of follow-up (95% CI 0.8-17.3). At the time of virological failure HIV-RNA resistance test was not achievable because of the low HIV-RNA peak reached (114 cp/ml).

**Figure 1:**
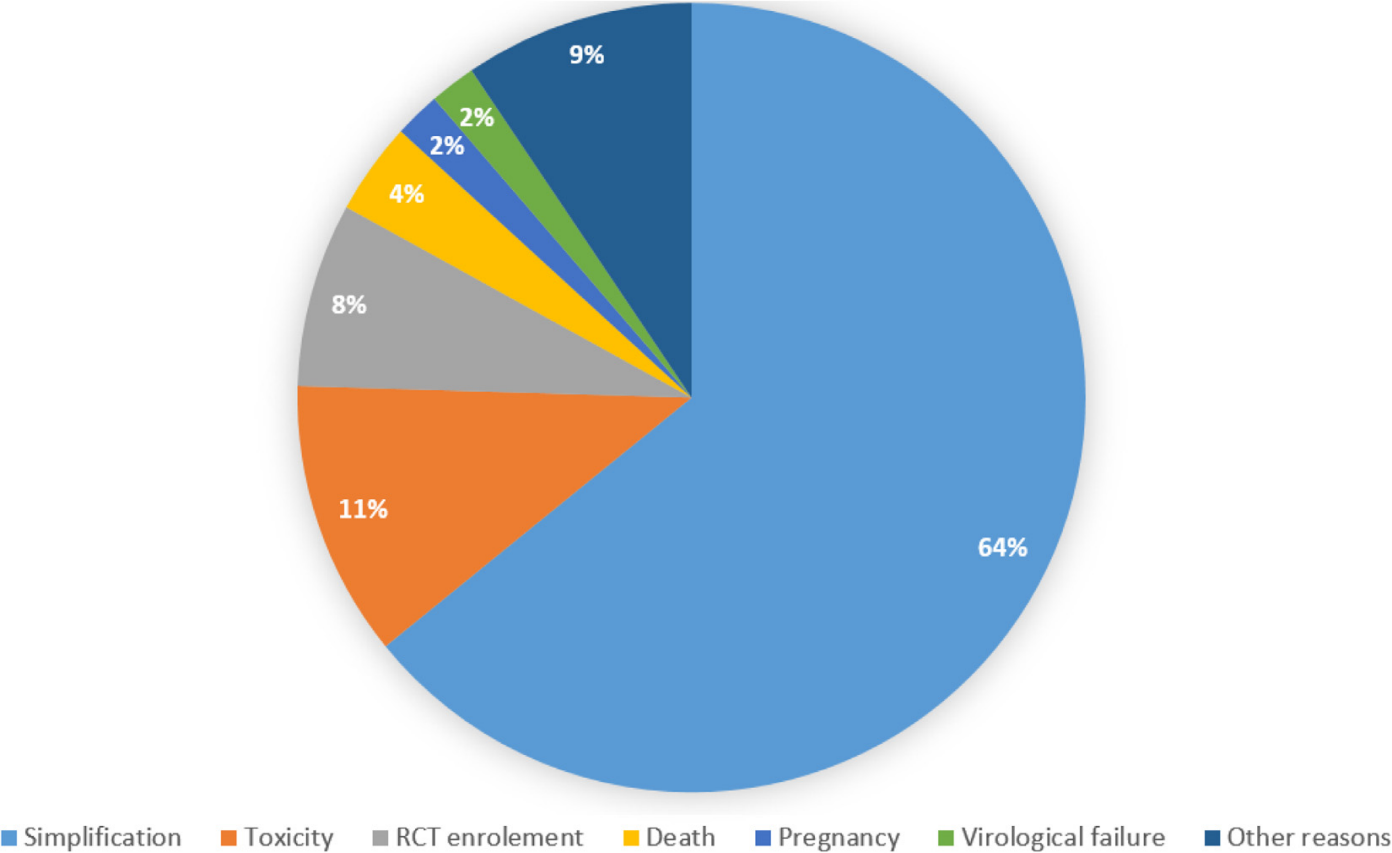
Reasons of tenofovir alafenamide/emtricitabine/bictegravir interruptions. RCT, randomized control trial.

**Figure 2:**
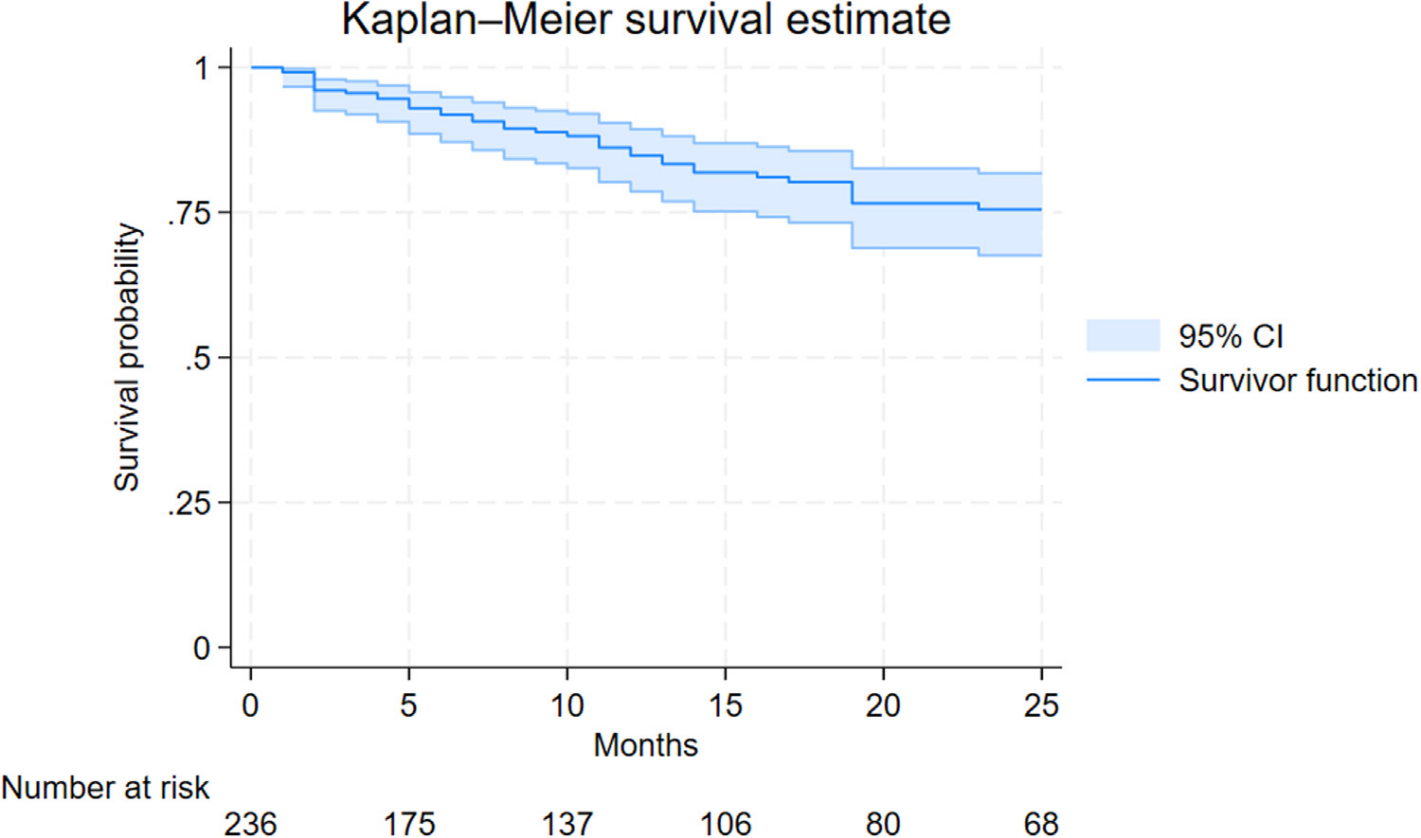
Durability of tenofovir alafenamide/emtricitabine/bictegravir estimated using the Kaplan-Meier survival function. CI, confidence interval.

**Figure 3:**
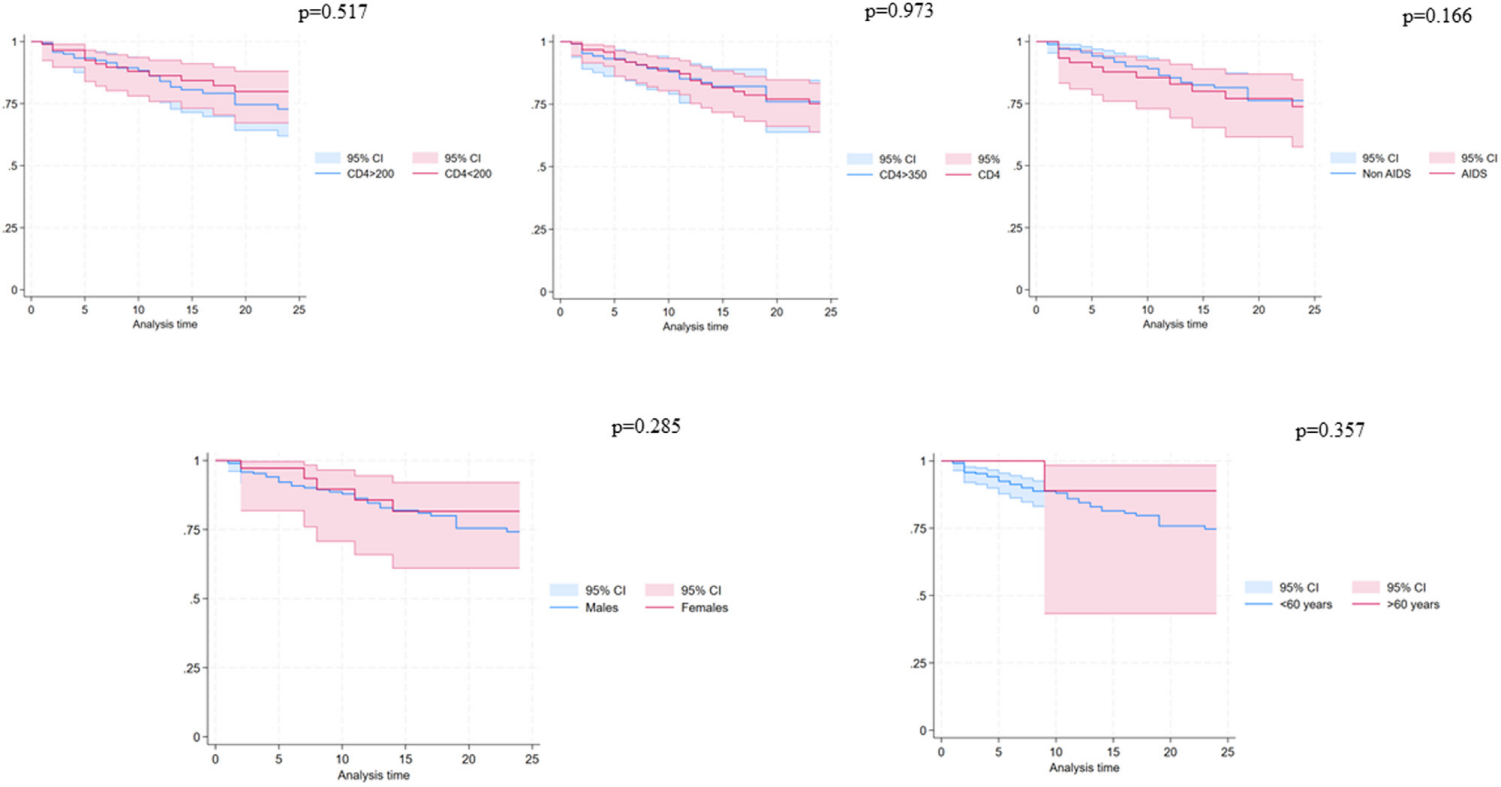
Durability of tenofovir alafenamide/emtricitabine/bictegravir estimated using the Kaplan-Meier survival function according to different characteristics at the time of treatment start. CD, clusters of differentiation; CI, confidence interval.

Fifty-two individuals (22%) interrupted TAF/FTC/BIC during the study period for reasons other than virological failure: 34 (14.4%) because of simplification, 6 (2.5%) because of toxicities, 4 (1.7%) for clinical trial enrollment, 2 (0.8%) died, 1 (0.4%) because of pregnancy and 5 (2.1%) for other reasons (Figure 1). The estimated durability of TAF/FTC/BIC at 12 and 24 months were 84.8% (95% CI 78.6-89.3%) and 75.5% (95% CI 67.6-82.6%), respectively (Figure 2). Kaplan-Meier survival plots according to different characteristics at the time of TAF/FTC/BIC start are depicted in Figure 3. No evidence for a difference in terms of durability was observed according to age (*P* = 0.357), biological sex (*P* = 0.285), CD4 cell count strata (< *vs* ≥350 cell/mm^3^ [*P* = 0.973] and < *vs* ≥200 [*P* = 0.517]) and AIDS presentation (*P* = 0.166).

## Discussion

In our study, we found that TAF/FTC/BIC as a first-line regimen in newly diagnosed PWH is effective and safe in a non-experimental context, characterized by a high prevalence of PWH presenting with advanced stages of infection.

The characteristics of our study population align with Italian national data [14] and approximate those of newly diagnosed PWH in Europe [15], with 53.4% of PWH presenting late versus 58.1% and 50.6%, respectively. The proportion of individuals with an AIDS-defining condition (27.4%) is also consistent with Italian [14] and European official data [15]. Therefore, our study population comprises individuals who require a rapid antiretroviral start according to current guidelines [1,2], often before resistance testing results are available [7,13]. In this clinical scenario, considering the low prevalence of transmitted drug resistance to INSTI in Europe [18], the use of a second-generation INSTI provides the benefit of a high genetic barrier to resistance [[3], [4], [5], [6], [7]] while maximizing the chances of virological effectiveness. Additionally, TAF/FTC/BIC offers the advantage of single-tablet co-formulation, providing benefits in terms of adherence [19], combined with an optimal pharmacodynamic profile and a low potential for drug-to-drug interactions [16]. This versatility of the regimen could explain the low incidence of protocol-defined virological failure which is in line with registration trials in newly diagnosed PWH in which no documentation of emergent resistance to the regimen was documented [3,5].

These characteristics together could explain the high durability observed in our study, with 84.8% of patients remaining on therapy at 12 months and 75.5% at 24 months. With no evidence of an effect in terms of treatment discontinuation because of low CD4 cell count or AIDS at the time of TAF/FTC/BIC. This finding is comparable to that reported in other cohort studies. For instance, in another multicenter Italian cohort, the durability in newly diagnosed PWH at 12 and 24 months was 90% (95% CI 87.8-91.9%) and 79.6% (95% CI 76.1-82.7%), respectively [11]. Slightly higher durability rates were observed in other observational sponsored studies conducted in Europe and Canada (97% at 12 months) [20]. In a large sponsored multicenter prospective study, at 24 months 400/420 (95%) treatment naive PWH enrolled were still in TAF/FTC/BIC since treatment started [21]. In the setting of late presentation (CD4 cell count <200 cell/mm^3^ or AIDS), a recent study conducted in Cina showed a high 48-week virological effectiveness comparable to our study with 93.8% (122/130) of the patients who achieved HIV-RNA levels <50 copies/ml without any TAF/FTC/BIC treatment discontinuation [22]. Moreover, in another Italian study with a long follow-up, the probability of remaining free from virological failure of TAF/FTC/BIC at 144 weeks was 95.2% [23].

The safety and tolerability of the TAF/FTC/BIC regimen were confirmed by our data, with only 2.5% of patients discontinuing treatment because of toxicity. This rate of discontinuation because of toxicity is comparable to that observed in other observational studies [11,20,24] and is expected considering the safety data from RTCs [[3], [4], [5], [6], [7]]. Most of the interruptions occurred for simplification reasons, as observed in other observational studies.

### Study limitations

Our study has several limitations. First, the monocentric study design limits the generalizability to other different settings. Second, in an observational setting, the choice of regimen may be influenced by individual patient characteristics and thus the results are prone to selection bias. Third, we only assessed adverse events that led to treatment discontinuation, not all possible adverse events. In the end, the absence of a control group did not allow us to estimate the effectiveness of this regimen when compared to the other available first-line regimens. In particular, because we do not have planned to compare treatment outcomes with other antiretroviral treatments, we do not implement a specification of target trial emulation which is now considered the standard when comparing treatment outcomes to inform clinical decisions as recommended by the European Medical Agency when RCTs on the topic are lacking [25].

Nevertheless, our study draws information from a large cohort of PWH treated in a non-experimental context characterized by a high proportion of individuals presenting late and with advanced disease.

## Conclusion

In our cohort of newly diagnosed PWH, TAF/FTC/BIC showed good durability up to 75% after 2 years since starting the treatment. Few interruptions appeared to be related to drug toxicities and a low rate of virological failure was observed although a high proportion of PWH who presented late or with an AIDS-defining condition.

## Declarations of competing interests

Andrea Giacomelli reports consulting fees from Mylan and Jansen; payment or honoraria for lectures, presentations, speakers’ bureaus, manuscript writing, or educational events from Gilead and ViiV; payment for expert testimony from Jansen; support for attending meetings and/or travel from Gilead, ViiV, and MSD. MVC has reports payment or honoraria for presentations, manuscript writing, or educational events from MSD, ViiV, Gilead, and Janssen-Cilag, DM received grants and fees for the speaker bureau, and CME activities from ViiV Healthcare, Merck & Co. Inc., Gilead Science Inc., and Viatris Inc.; fees for advisory boards from Johnson & Johnson and Gilead Science Inc., and non-financial educational support from Gilead Sciences Inc. and ViiV Healthcare., CG has received personal fees from MSD, ViiV, Gilead, and Janseen Cilag, outside the submitted work. S. A. reports payment or honoraria for lectures, presentations, speakers’ bureaus, manuscript writing, or educational events from Pfizer, and support for attending meetings and/or travel from Pfizer and MSD. Andrea Gori reports grants or contracts from ViiV, Bristol-Myers Squibb, and Gilead; consulting fees from ViiV Healthcare, Gilead, Janssen-Cilag, Merck Sharp & Dohme, Bristol-Myers Squibb, Pfizer, and Novartis; payment or honoraria for lectures, presentations, speakers bureaus, manuscript writing or educational events from ViiV Healthcare, Gilead, Janssen-Cilag, Merck Sharp & Dohme, Bristol-Myers Squibb, Pfizer, and Novartis; support for attending meetings and/or travel from ViiVHealthcare, Gilead, Janssen-Cilag, Merck Sharp & Dohme, Bristol-Myers Squibb, Pfizer, and Novartis. GC, SR, FS, GP, MLC, CF, and ALR have nothing to declare.
